# Estimating the epidemiology of chronic Hepatitis B Virus (HBV) infection in the UK: what do we know and what are we missing?

**DOI:** 10.12688/wellcomeopenres.17941.1

**Published:** 2022-08-08

**Authors:** Cori Campbell, Tingyan Wang, Rebekah Burrow, Sema Mandal, Julia Hippisley-Cox, Eleanor Barnes, Philippa C Matthews

**Affiliations:** 1 Nuffield Department of Medicine, University of Oxford, Oxford, OX1 3XY, UK; 2 Oxford Biomedical Research Centre, Oxford, UK; 3 QResearch / Nuffield Dept of Primary Care, Oxford, UK; 4 Blood safety, Hepatitis, STI & HIV Division,, UK Health Security Agency, London, UK; 5 National Institute for Health Research Health Protection Unit, London, UK; 6 Department of Hepatology, Oxford University Hospitals, John Radcliffe Hospital, Oxford, UK; 7 The Francis Crick Institute, London, UK; 8 Division of Infection and Immunity, University College London, London, UK; 9 Department of Infectious Diseases, University College London Hospitals NHS Trust, London, UK

**Keywords:** hepatitis B virus, HBV, prevalence, epidemiology

## Abstract

**Background: **HBV is the leading global cause of cirrhosis and primary liver cancer. However, the UK HBV population has not been well characterised, and estimates of UK HBV prevalence and/or incidence vary widely between sources. We summarised datasets that are available to represent UK CHB epidemiology, considering differences between sources, and discussing deficiencies in current estimates.

**Methods: **We searched for estimates of CHB case numbers in the UK (incorporating incidence and/or prevalence-like data) across a range of available sources, including UK-wide reports from government bodies, publications from independent bodies (including medical charities and non-governmental organisations) and articles in peer-reviewed scientific journals to collate estimated positivity rates. An alternative proxy for population prevalence was obtained via the UK antenatal screening programme which achieves over 95% coverage of pregnant women.

**Results: **We identified six CHB case number estimates, of which three reported information concerning population subgroups, including number of infected individuals across age, sex and ethnicity categories. Estimates among sources reporting prevalence varied from 0.27% to 0.73%, congruent with an estimated antenatal CHB prevalence of <0.5%.

**Discussion**: Estimates varied by sources of error, bias and missingness, data linkage, and substantial “blind spots” in consistent testing and registration of HBV diagnoses. The HBV burden in the UK is likely to be concentrated in vulnerable populations who may not be well represented in existing datasets including those experiencing socioeconomic deprivation, ethnic minorities, people experiencing homelessness and people born in high-prevalence countries. Together, these factors could lead to either under- or over-estimation of overall prevalence, and additional efforts are required to provide estimates that best reflect the whole population.  Multi-parameter evidence synthesis and back-calculation model methods similar to those used to generate estimates of HCV ad HIV population-wide prevalence may be applicable to HBV.

## Introduction

Hepatitis B virus (HBV) is the leading global cause of cirrhosis, and of primary liver cancer incidence and mortality ^1, 2^. Nearly 300 million individuals worldwide are estimated to be living with chronic HBV (CHB) infection. Risks of complications and death are mitigated by screening to detect cases of infection, clinical monitoring of chronic infection (including liver cancer surveillance in high-risk cases), and antiviral therapy in those who meet treatment criteria ^3^.

The United Kingdom (UK) is regarded as a low-prevalence setting for CHB ^3^. However, the attributable disease burden may be substantial in specific population subgroups including people who inject drugs, the prison population, people experiencing homelessness, and individuals belonging to minority ethnic groups and born in countries where the prevalence of CHB is higher ^4^. Thus, CHB is concentrated in potentially vulnerable and/or disadvantaged population subgroups.

Epidemiological characterisation of the UK CHB population has been limited, with no central registry of infected persons. Existing data may primarily reflect new diagnoses (a combination of incident acute infection and new diagnoses of chronic infection), but caution is needed in making inferences about prevalence. Accurate estimation of prevalence is challenging, because complete HBV data are not likely to be well captured by large-scale electronic health record (EHR) databases for either primary or secondary care ^5^, as many CHB cases remain untested and therefore undiagnosed.

The World Health Organization (WHO) has set targets for viral hepatitis elimination within its Sustainable Development Goals for 2030. The Global Health Sector Strategy on Viral Hepatitis ^6^ identifies specific goals, including diagnosis in 90% of chronic infections, 90% reduction in incidence of chronic infection, and 80% treatment coverage in those eligible. High quality epidemiological data are therefore crucial to focus and measure progress, inform policy and interventions, reduce inequities and underpin resource allocation. We herein summarise datasets that are available to represent UK CHB epidemiology, consider differences between sources, and discuss deficiencies in current estimates.

## Methods

We searched for estimates of CHB case numbers in the UK (incorporating incidence and/or prevalence-like data) across a range of available sources. We included UK-wide reports from government bodies, publications from independent bodies (including medical charities and non-governmental organisations) and articles in peer-reviewed scientific journals. We present positivity rates from each respective data source, but caution that these estimates are not representative of the true UK-wide population prevalence. Details of study samples/denominator are provided. The Office for National Statistics (ONS) provides UK population estimates as a point of reference for the overall denominator ^7^.

We also utilised data from the UK primary care database QResearch, which contains over 35 million patient records from more than 1800 individual practices ^7^. QResearch was established in 2002 and contains anonymised individual-level patient EHR. Data are collected prospectively and are linked to hospital episode statistics (HES), National Cancer Registration Analysis Service (NCRAS) and ONS mortality data. QResearch ethics approval is with East Midlands-Derby Research Ethics Committee (reference 18/EM/0400).’

We identified individuals in the QResearch (version 44) database who had a record of a diagnostic Systemised Nomenclature of Medicine (SNOMED)/Read or International Classification of Disease (ICD) code indicative of CHB, or who had a history of ≥1 hepatitis B surface antigen (HBsAg) or viral load (VL) measurement. From this sample we identified individuals between 01 January 1999 and 31 December 2019, age ≥18 years with CHB, defined as: i) record of a diagnostic SNOMED/Read code indicating CHB; or ii) record of a diagnostic ICD-9 or -10 code indicating CHB; and/or iii) Presence of HBsAg or VL on ≥2 recordings ≥6 months apart. The characteristics of HBV infection in the cases we identified are further described elsewhere ^7^.

We have also drawn on findings from a similar investigation previously undertaken in the Clinical Practice Research Datalink (CPRD) ^8^, which is another UK primary care database containing EHRs for over 16 million patients. This previous investigation identified CHB individuals from patients registered in the database between 2000 and 2015.

This article can be found on medRxiv ^9^.

## Results and discussion

UK data for CHB epidemiology are summarised in Table 1. Three of six estimates report information concerning population demographics, including number of infected individuals across age, sex and ethnicity categories. Among sources setting out to report prevalence, estimates varied from 0.27% (British Liver Trust / Department of Health and Social Care (DHSC) 2002 estimate) to 0.73% (estimate by the Polaris Institute). An alterantive proxy for population prevalence is obtained via the UK antenatal screening programme, which achieves over 95% coverage of every pregnant woman annually (approx. 700,000 women in the UK), with a CHB prevalence of <0.5% ^10^. Differences between sources highlights varied sources of error, bias and missingness, problems with data linkage, and substantial “blind spots” in consistent testing and registration of HBV diagnoses.

**Table 1.** Prevalence estimates for chronic hepatitis B virus (HBV) infection in sub-populations in the United Kingdom.

Estimate source	Population and/or data source (study period/year)	Sources and citation(s)	Estimate type	Denominator (population tested)	Number of adults testing positive for HBV infection	Percentage infected with HBV in study/ survey population (%)	Information on relevant population characteristics ^†^
**PHE / UKHSA**	Individuals tested for HBsAg in 19 participating laboratories (excludes testing in antenatal services and reference lab testing; deduplicated using identifiers where available) (2020)	Sentinel surveillance of BBV testing ^11^	Positivity amongst those tested (diagnosed prevalence)	302,135	2611	0.86%	Yes: information by ethnicity, sex, age, region
**Polaris ** **Observatory** ^12^	Modelling study based on literature review and expert interviews (2016)	Razavi-Shearer *et al.,* 2018 ^13^	UK HBV population prevalence	2016 UK population of 65,648,000 ^14^	440,000	0.73%	No
**PHE Infectious ** **Diseases in ** **Pregnancy ** **Screening ** **Programme**	Pregnant women attending antenatal services in England (April 2019-March 2020)	Antenatal screening standards data report 1 April 2019 – 31 March 2020 ^10, 15^	Antenatal population prevalence	Pregnant women attending antenatal services 661,281 (screening coverage of 99.8%)	2493	0.38%	No
**BLT** ^16^ *	Source unavailable (likely 2002) ^§^	NICE guidelines ^17, 18^**	Number of people in the UK positive for HBV - prevalence	Not clearly stated – likely 2002 UK population of 59,365,677 ^19^	180,000	0.27%	No
**CPRD**	CPRD primary care database consisting of EHRs from >16 million patients (2000 to 2015)	Ferreira *et al.* ^8^	Number of CHB diagnoses in database	CPRD subset of EHRs from 4.4 million patients ^¶^	3927	0.09%	Yes
**QResearch ** **cohort** ^20^	QResearch primary care database (1999 to 2019)	CCCC-UK cohort ^7, 20^	Number of CHB diagnoses in database	QResearch database subset of EHRs from 16.9 million patients ^5^	8039	0.05%	Yes

HBV, Hepatitis B virus; UKHSA, United Kingdom Health Security Agency; PHE, Public Health England; PWID, people who inject drugs; BLT, British Liver Trust; NICE, National Institute for Health and Care Excellence; CPRD, Clinical Practice Research Datalink; CHB chronic Hepatitis B virus infection; EHR, electronic health record; CCCC, Characteristics of Chronic Hepatitis B associated with Cirrhosis and Cancer. * The British Liver Trust published a HBV report in 2017 stating that approximately 180,000 people live with chronic HBV infection in the UK ^16^. This report cited a governmental migrant health guide citing this prevalence estimate ^17^. The source which this government report cited was a National Institute for Health and Care Excellence (NICE) testing guideline which stated this prevalence estimate and dated it to 2002 ^18^. † May include but is not limited to: age, sex, ethnicity and socioeconomic status. ‡ Sources include “laboratory data and sentinel surveillance data” ^11^. § Department of Health 2002 report cited ^21^ is unavailable for download. No clear methodology reported ¶ Subset of total (16.9 million) patients in CPRD with valid EHR information and temporality who were acceptable for research ^8^.

As HBV is a notifiable disease in the UK, the UK Health Security Agency, UKHSA (previously Public Health England, PHE), has a comprehensive surveillance system for monitoring burden of CHB, by monitoring testing, blood donor screening and diagnoses across the care pathway. This incorporates data from diagnoses through to outcomes, (including end-stage liver disease, transplantation, liver cancer and deaths) using laboratory testing surveillance, hospital activity datasets and registries (sentinel surveillance of blood-borne virus (BBV) testing, new laboratory diagnoses, hospital episode statistics, ONS cancer and deaths registries). However, these data have not yet been combined and incorporated in a statistical model to estimate prevalence. Sentinel surveillance captures testing in community, primary care and secondary care settings across a network of laboratories covering approximately 40% of the general population of England ^11^. This likely gives the best estimate of diagnosed prevalence among a tested population, but because it combines acute incident infections and new diagnoses of pre-existing chronic infection, incidence and prevalence cannot be disaggregated.

The majority of diagnostic data are generated through testing individuals with risk factors for HBV infection or evidence of liver disease, and are therefore at risk of over-estimating true prevalence. However, no existing estimates factor in the undiagnosed burden, which represents the majority of people living with HBV infection (the WHO estimates that only 10.5% of people with CHB are aware of their infection status ^3^). Furthermore, the highest prevalence of CHB is in groups for whom provision of healthcare is inadequate, and/or access to healthcare is challenging (including migrants, sex-workers, prisoners, and people experiencing homelessness), so overall there are still many gaps in the data, and it is most likely that estimates using primary care datasets considerably underestimate the true burden. In contrast, prevalence or test positivity among those accepting risk-based testing (as captured in laboratory testing surveillance) likely overestimates the overall population prevalence.

While UKHSA surveillance data may include some demographic characteristics (age, sex, postcode for deprivation), unless linked to other healthcare datasets, they typically lack more detailed clinical and demographic indicators (for example, measures of deprivation, lifestyle factors, assessment of liver disease, and HBV treatment coverage) which are needed to characterise the infected population. In constrast, EHR databases (such as CPRD and QResearch) have the advantage of collecting relevant demographic and clinical metadata which are not captured by UKHSA. However, linkage across data sources is disaggregated, and thereby each EHR-based estimate misses a portion of the infected population. For example, primary care data may not reflect testing conducted in secondary care ^22^, blood safety (transfusion/transplantation) and laboratory data generated by other services, while secondary care data are typically only reliable for the sub-population enrolled in consistent hospital follow-up. Poor data flow between diagnostic testing and EHR reflect a low clinical follow-up rate following a positive HBsAg test. This limited linkage to care reflects how services may not provide well for the CHB population, with gaps in referral pathways, inadequate communication and education (including translation services), and failures to deliver services to marginalised communities. Therefore, EHR databases offer the potential to characterise a subset of those infected with HBV, but do not currently generate a picture that is generalisable to the wider infected population, and cannot on their own be used to estimate prevalence.

Prevalence estimates for Hepatitis C virus (HCV) ^23^ and human immunodeficiency virus (HIV) ^24^ have recently been generated using multi-parameter evidence synthesis and back-calculation models. Similar modelling approaches to produce estimates of HBV incidence and prevalence in the UK are warranted. Enhanced investment is needed to support the establishment of national registries with robust centralised data linkage between sources including national laboratory surveillance systems of BBV testing and new diagnoses, and thus determine which population subgroups are bearing the majority of the HBV disease burden. This will inform prevalence modelling and provide an evidence base for delivery of appropriate resources and interventions, and to benchmark progress towards elimination targets.

**Summary box: Recommendations for the generation of enhanced insights into national CHB caseload**

- Expansion of systematic screening, including opportunistic approaches (sexual health, antenatal, emergency medicine, people born in high-prevalence settings).
- Improved centralised data linkage between services, including laboratory records, blood and transplant services, primary and secondary care, supported by collection of metadata.
- Disaggregation of incidence/prevalence data where possible at source.
- Establishment of regional and/or national registries to collate linked data for HBV infection at a population level and within high risk groups.
- Mathematical modelling to optimise use of existing data to generate incidence and prevalence estimates, identify systematic data gaps, refine allocation of resources and predict progress towards elimination targets.

## Ethics approval

QResearch ethics approval is with East Midlands-Derby Research Ethics Committee (reference 18/EM/0400).

## Data availability

Only CC, TW, RB and JH-C have access to the QResearch individual-level patient data in order to ensure confidentiality of personal and health information, in accordance with the relevant licence agreements. QReseearch data access is according to the information on the QResearch website ( www.qresearch.org).
