# Supplementary material for: Estimating the epidemiology of chronic Hepatitis B Virus (HBV) infection in the UK: what do we know and what are we missing?
Source: Wellcome Open Res. 2023 Mar 9;7:203. Originally published 2022 Aug 8. [Version 2] doi: 10.12688/wellcomeopenres.17941.2 (PMC9975428; doi:10.12688/wellcomeopenres.17941.2)
Supplement: Peer review history

# Peer review: Reviewer response for version 2

**DOI:** 10.21956/wellcomeopenres.21210.r55464

**Published:** 2023-03-15

**Authors:** Anders Boyd

**Affiliations:** 1 HIV Monitoring Foundation, Amsterdam, The Netherlands

All concerns were addressed.

Is the work clearly and accurately presented and does it cite the current literature?

Yes

If applicable, is the statistical analysis and its interpretation appropriate?

Not applicable

Are all the source data underlying the results available to ensure full reproducibility?

No source data required

Is the study design appropriate and is the work technically sound?

Partly

Are the conclusions drawn adequately supported by the results?

Yes

Are sufficient details of methods and analysis provided to allow replication by others?

Partly

Reviewer Expertise:

HBV, HIV, STI, epidemiology, biostatistics

I confirm that I have read this submission and believe that I have an appropriate level of expertise to confirm that it is of an acceptable scientific standard.

# Peer review: Reviewer response for version 2

**DOI:** 10.21956/wellcomeopenres.21210.r55465

**Published:** 2023-03-10

**Authors:** Lung-Yi Mak

**Affiliations:** 1 Department of Medicine, Queen Mary Hospital, The University of Hong Kong, Hong Kong, China

The authors have addressed the salient comments.

Is the work clearly and accurately presented and does it cite the current literature?

Partly

If applicable, is the statistical analysis and its interpretation appropriate?

Not applicable

Are all the source data underlying the results available to ensure full reproducibility?

Yes

Is the study design appropriate and is the work technically sound?

Yes

Are the conclusions drawn adequately supported by the results?

Partly

Are sufficient details of methods and analysis provided to allow replication by others?

Yes

Reviewer Expertise:

chronic hepatitis B infection, clinical liver disease

I confirm that I have read this submission and believe that I have an appropriate level of expertise to confirm that it is of an acceptable scientific standard.

# Peer review: Reviewer response for version 1

**DOI:** 10.21956/wellcomeopenres.19882.r53572

**Published:** 2023-01-11

**Authors:** Anders Boyd

**Affiliations:** 1 HIV Monitoring Foundation, Amsterdam, The Netherlands

In their manuscript, Campbell et al describe the available sources used to estimate the case numbers of hepatitis B virus (HBV) in the United Kingdom, while highlighting the major gaps in understanding HBV epidemiology in the country. The details on the data sources retrieved are clear, along with their limitations. I found the discussion to be particularly helpful. Nevertheless, there are some issues worth addressing.

 I appreciate that this is a narrative review, yet the information on what was searched (i.e., which keywords), in which locations (i.e., with which search engines/sources) and when (i.e., years in which databases were allowed) is unclear. It would also be helpful to include some notion of which databases were included and excluded. For instance, it seems that many small epidemiological studies intended to find prevalence or incidence in very specific demographic populations could be missed (which could help address some of the gaps stated by the authors). Including this information would help guide the reader as to how the authors arrived at their selection.

 I also very much appreciate the summary box, which includes recommendations on how to achieve national CHB caseloads. But given the low prevalence of HBV and higher prevalence in specific key populations, would it make sense to aim for a national CHB caseload estimate? The aim of these recommendations should more reflect what the authors stated in their title, towards understanding the epidemiology of HBV in the United Kingdom. It would also be helpful to include a column in this box with the gap(s) in knowledge corresponding to the given recommendation, so the reader can identify the limitations of existing data (and hopefully avoid it in the future).

 Finally, it is unclear why prevalence estimates were given in the results, or even why this was examined in the first place if the aim was to discuss data sources to understand HBV epidemiology (i.e., data sources that have an estimate of HBV prevalence). I would suggest that the authors make it clear why this statistic was included, possibly by adding it as a secondary objective.

 Minor comments:

 - Abstract. “…(incorporating incidence and/or prevalence like data)…” is ambiguous. Please clarify.

 - Abstract. “sources of error” refers to what exactly? This term could be deleted as it seems that bias was more the focal point of the discussion.

 - Abstract. Although I agree with the sentence, “Multi-parameter evidence … may be applicable to HBV.” – this does not really seem appropriate for the abstract (as the result backing this argument is missing in the Abstract).

 - Introduction. The information on ERH is probably more appropriate for the discussion. The third paragraph in the introduction should focus not only on the limited data, but also the lack of summary on HBV data in the UK. This information could be used to help policy makers understand what exactly are the gaps in knowledge.

 - Introduction. It should be stated clearly in the aims that this is a narrative review (for readers who may confuse this with a systematic review and meta-analysis).

Is the work clearly and accurately presented and does it cite the current literature?

Yes

If applicable, is the statistical analysis and its interpretation appropriate?

Not applicable

Are all the source data underlying the results available to ensure full reproducibility?

No source data required

Is the study design appropriate and is the work technically sound?

Partly

Are the conclusions drawn adequately supported by the results?

Yes

Are sufficient details of methods and analysis provided to allow replication by others?

Partly

Reviewer Expertise:

HBV, HIV, STI, epidemiology, biostatistics

I confirm that I have read this submission and believe that I have an appropriate level of expertise to confirm that it is of an acceptable scientific standard, however I have significant reservations, as outlined above.

# Peer review: Reviewer response for version 1

**DOI:** 10.21956/wellcomeopenres.19882.r53567

**Published:** 2023-01-03

**Authors:** Jonggi Choi

**Affiliations:** 1 Department of Gastroenterology, Liver Center, Asan Medical Center, University of Ulsan College of Medicine, Seoul, South Korea

The authors extensively reviewed the current data regarding the chronic hepatitis B epidemiology in the UK. The authors also acknowledged limitations on current data about chronic hepatitis B due to the limited source. Therefore, it requires a broad spectrum study to cover entire population to help guide public health policy and control transmittable disease.

 I have no further comment on this manuscript.

Is the work clearly and accurately presented and does it cite the current literature?

Yes

If applicable, is the statistical analysis and its interpretation appropriate?

Yes

Are all the source data underlying the results available to ensure full reproducibility?

Yes

Is the study design appropriate and is the work technically sound?

Yes

Are the conclusions drawn adequately supported by the results?

Yes

Are sufficient details of methods and analysis provided to allow replication by others?

Yes

Reviewer Expertise:

Hepatology

I confirm that I have read this submission and believe that I have an appropriate level of expertise to confirm that it is of an acceptable scientific standard.

# Peer review: Reviewer response for version 1

**DOI:** 10.21956/wellcomeopenres.19882.r53571

**Published:** 2022-12-13

**Authors:** Lung-Yi Mak

**Affiliations:** 1 Department of Medicine, Queen Mary Hospital, The University of Hong Kong, Hong Kong, China

This is an extremely important piece of work to estimate the epidemiology of HBsAg+ seropositivity in the UK. The authors summarized data from 6 sources, leading to a crude estimate for HBsAg seropositivity ranging from 0.27-0.73%. The following points should be addressed to further enrich the scientific contents and provide some directions for further research:

 1. Ongoing pilot programs of universal HBV testing at Emergency Departments are being conducted in some NHS Trusts, especially after the COVID-19 pandemic. Would there be any chance that updated data from these pilot programs might have been recently reported and can be included in this piece?

 2. Regional differences within the country, or even to a smaller scale, within a city, should also be appreciated. For instance, East London is likely having a much higher prevalence of HBsAg+ than the West/ North side of the city. While population-based data is very important, targeting high-risk groups (as the authors also addressed) is equally crucial to inform healthcare strategies such as resource allocation. This is also relevant to UK in view of the low overall prevalence of HBsAg (<2%, according to data presented), and population-based screening may not be as cost-effective as other highly endemic regions. Although the CDC has updated the recommendations since 2022 to screen for HBV infection for at least once in the lifetime for all adults, the actual implementation of such approach will highly depend on the resources available. Therefore, it would be helpful if there is data for HBsAg seroprevalence in the high-risk groups; and in addition to that addressed in this article (sexual health, antenatal, emergency medicine, people born in high-prevalence setting), regions that are traditionally considered to be impoverished areas or known to harbour a relatively high proportion of immigrants, should be the target groups for such opportunistic approaches.

 3. Would there be any overlap between the laboratory-identified HBsAg+ cases from UKHSA with other data sources? (i.e., the primary care database QResearch, CPRD primary care database and the antenatal screening programme)

 4. Risk of bias and sampling error is well acknowledged – the authors may also include data from the NHS Blood and Transplant study published in Transfusion 2021 which reported a seroprevalence of HBsAg+ being 6.9/ 100,000 donors between 2009-2018.

Is the work clearly and accurately presented and does it cite the current literature?

Partly

If applicable, is the statistical analysis and its interpretation appropriate?

Not applicable

Are all the source data underlying the results available to ensure full reproducibility?

Yes

Is the study design appropriate and is the work technically sound?

Yes

Are the conclusions drawn adequately supported by the results?

Partly

Are sufficient details of methods and analysis provided to allow replication by others?

Yes

Reviewer Expertise:

chronic hepatitis B infection, clinical liver disease

I confirm that I have read this submission and believe that I have an appropriate level of expertise to confirm that it is of an acceptable scientific standard, however I have significant reservations, as outlined above.
